# Arthrectomy of the Knee Joint for Tuberculous Disease

**Published:** 1900-01-27

**Authors:** 


### ARTHRECTOMY OF THE KNEE JOINT FOR TUBERCULOUS DISEASE.

At the last meeting of the Clinical Society a remarkably successful case of arthrectômy for tuberculous disease of the knee joint was shown by Mr. Frederick Eve. The patient was a girl 13 years of age, who had been admitted to the Evelina Hospital, with all the usual signs of strumous disease of the knee. As no improvement resulted after six weeks' treatment by immobilisation and Scott's dressing, Mr. Eve performed a complete arthrectomy, removing the whole of the thickened synovial membrane. A thin slice was also removed from the extremity of the internal condyle where the cartilage was destroyed. The lateral and crucial ligaments were carefully preserved, one of the latter which had to be cut through being afterwards refixed to the bone. The semilunar cartilages, however, were removed. The incision was made over the patella which was divided, the capsule being opened by lateral incisions avoiding the lateral ligaments. After the operation the incision in the capsule was carefully sewn up with silk sutures. The case ran an aseptic course, and a month after the operation the adhesions were broken down under chloroform, and since then on two or three occasions passive movement had been performed, also under anæsthetics. The result was that the limb was quite straight and firm, the knee could be flexed to a right angle, and the patient walked firmly and evenly upon it. Mr. Eve was warmly congratulated upon the result. In reply, he said that the case had been distinctly of the sub-acute variety of tuberculous disease of the joint, one in which fibrous tissue was undoubtedly forming in the synovial membrane.

